# Supplementary material for: Local genetic context shapes the function of a gene regulatory network
Source: eLife. 2021 Mar 8;10:e65993. doi: 10.7554/eLife.65993 (PMC7968929; doi:10.7554/eLife.65993)
Supplement: Supplementary file 1. [file elife-65993-supp1.docx]

**Supplementary File 1.** Strains used in this study.

| **Name** | **Genotype^a^** | **Source** |
| --- | --- | --- |
| DH5α | F^–^ *,Φ80lacZ*Δ*M15,* Δ*(lacZYA-argF), U169, recA1, endA1, hsdR17 (rK–, mK+), phoA supE44, thi-1, gyrA96, relA1, λ^–^* | Laboratory stock |
| DH5α λ*pir*+ | F^–^ *,Φ80lacZ*Δ*M15,* Δ*(lacZYA-argF), U169, recA1, endA1, hsdR17 (rK–, mK+), phoA supE44, thi-1, gyrA96, relA1, λpir^+^* | Laboratory stock |
| HG105 | MG1655 Δ*lacZYA* Δ*lacI* | (Garcia et al., 2011) |
| JW0336 | F^–^, Δ(*araD*-*araB*)567, Δ*lacZ*4787(::*rrnB*-3), Δ*lacI*785::kan, λ*^–^*, *rph*-1, Δ(*rhaD*-*rhaB*)568, *hsdR*514 | (Baba et al., 2006) |
| MG1655 | F-, λ^-^, *ilvG*-, *rfb*-50, *rph*-1 | Laboratory stock |
| TB201 | MG1655 att_P21_::P_R_-*yfp* | (Pleška et al., 2016) |
| Frag1B | F^–^, *lacZ82*(Am), λ*^–^*, *rha-4,* *thiE*, *gal-33, P_N25_/tetR placi^q^/lacI Sp^R^* | Laboratory stock |
| Frag1D | F^–^, *lacZ82*(Am), λ*^–^*, *rha-4,* *thiE*, *gal-33, P_N25_/tetR placi^q^/lacI Sp^R^*, Δ*recA* | Laboratory stock |
| ASE023 | MG1655 att_P21_::P_R_-*yfp* Δ*lacI*785 Δ*lacZ*4787::*rrnB*-3 | This study |
| ASE030 | MG1655 att_P21_::P_R_-*yfp* Δ*lacI*785 Δ*lacZ*4787::*rrnB*-3 Δ*recA* att_HK022_::P_lacO1_-*lacI* att_λ_::P_lacO1_-*tetR* *old*::P_tetO1_-*cI* Cam^R^ | This study |
| ASE031 | MG1655 att_P21_::P_R_-*yfp* Δ*lacI*785 Δ*lacZ*4787::*rrnB*-3 att_HK022_::P_LtetO1_-*cI*-P_LlacO1_-*tetR-*P_LlacO1_-*lacI* (CTL) | This study |
| ASE032 | MG1655 att_P21_::P_R_-*yfp* Δ*lacI*785 Δ*lacZ*4787::*rrnB*-3 att_HK022_::P_LlacO1_-*lacI*-P_LtetO1_-*cI-*P_LlacO1_-*tetR* (LCT) | This study |
| ASE033 | MG1655 att_P21_::P_R_-*yfp* Δ*lacI*785 Δ*lacZ*4787::*rrnB*-3 att_HK022_::P_LlacO1_-*tetR*-P_LlacO1_-*lacI-*P_LtetO1_-*cI* (TLC) | This study |
| ASE039 | MG1655 Δ*lacZYA* Δ*lacI* att_λ_::P_lac-131-410_-*yfp* | This study |
| ASE041 | MG1655 P*lac*::Cam^R^-FRT | This study |
| ASE046 | MG1655 Δ*lacZYA* Δ*lacI* att_λ_::P_lac-131-410_-*yfp* *fhlC*::*lacI* | This study |
| ASE047 | MG1655 Δ*lacZYA* Δ*lacI* att_λ_::P_lac-131-410_-*yfp yeaH::lacI* | This study |
| ASE048 | MG1655 Δ*lacZYA* Δ*lacI* att_λ_::P_lac-131-410_-*yfp asnT::lacI* | This study |
| KT131 | MG1655 att_P21_::P_R_-*yfp* Δ*lacI*785 Δ*lacZ*4787::*rrnB*-3 Δ*recA* | This study |
| KT132 | MG1655 Δ*lacI*785 Δ*lacZ*4787::*rrnB*-3 Δ*recA* | This study |

^a^ Amp – ampicillin resistance, Kan – kanamycin resistance, Sp – spectinomycin resistance, Cam – chloramphenicol resistance
