# Supplementary material for: Local genetic context shapes the function of a gene regulatory network
Source: eLife. 2021 Mar 8;10:e65993. doi: 10.7554/eLife.65993 (PMC7968929; doi:10.7554/eLife.65993)
Supplement: Supplementary file 2. [file elife-65993-supp2.docx]

**Supplementary File 2. Oligonucleotides used in this study.**

| **Name** | **Sequence (5’-3’)^a^** | **Use** |
| --- | --- | --- |
| AS096 | GTACCTGCAGGACGTCAGTACTGCCA*CGG*AGGC**ATACGCAAACCGCCTCTCCC** | *mCherry* up |
| AS097 | GTACCCCGGGAGATCTGTCGACGCCT*AGG*TGGC**CTACTAGTATATAAACGCAG** | *mCherry* down |
| AS098 | ATTCGCCA*CGG*AGGCA**CCTTTCGTCTTCACC** | Fragment 1F, up**^b^** |
| AS099 | GACAGCCT*GAA*TGGC**TCTAGGGCGGCGGATTTG** | Fragment 1F, down**^b^** |
| AS100 | ATTCGCCA*TTC*AGGCA**CCTTTCGTCTTCACC** | Fragment 2F, up**^b^** |
| AS101 | GACAGCCT*CAC*TGGC**TCTAGGGCGGCGGATTTG** | Fragment 2F, down**^b^** |
| AS102 | ATTCGCCA*GTG*AGGCA**CCTTTCGTCTTCACC** | Fragment 3F, up**^b^** |
| AS103 | GACAGCCT*AGG*TGGC**TCTAGGGCGGCGGATTTG** | Fragment 3F, down**^b^** |
| AS104 | **TTCACCTCGAGAATTGTGAGC** | P_LlacO1_-*lacI/tetR*, up |
| AS105 | GACA**TCTAGATTAAGCTGCTAAAGCGTAG** | P_LlacO1_-*lacI/tetR*, down |
| AS106 | **TTCACCTCGAGTCCCTATCAG** | P_LtetO1_-*cI*, up |
| AS107 | **GGATCCTCTAGATCAAGCTGC** | P_LtetO1_-*cI*, down |
| AS129 | ATTCGCCA*CGG*AGGC**TCTAGGGCGGCGGATTTG** | Fragment 1R, up**^b^** |
| AS130 | GACAGCCT*GAA*TGGCT**CCTTTCGTCTTCACC** | Fragment 1R, down**^b^** |
| AS131 | ATTCGCCA*TTC*AGGC**TCTAGGGCGGCGGATTTG** | Fragment 2R, up**^b^** |
| AS132 | GACAGCC*TCA*CTGGCTC**CTTTCGTCTTCACC** | Fragment 2R, down**^b^** |
| AS133 | ATTCGCCA*GTG*AGGC**TCTAGGGCGGCGGATTTG** | Fragment 3R, up**^b^** |
| AS134 | GACAGCCT*AGG*TGGCTC**CTTTCGTCTTCACC** | Fragment 3R, down**^b^** |
| AS135 | GCATGAATTC**GGCTGTTCTGGTGTTGCTAG** | pZS*2 backbone, up |
| AS136 | GTCAAGTACT**CTCGAGGTGAAGACGAAAGG** | pZS*2 backbone, down |
| AS142 | CTAGAAAAAGCCTCCGACCGGAGGCTTTTGT | T*tonB*, fwd |
| AS143 | CTAGACAAAAGCCTCCGGTCGGAGGCTTTTT | T*tonB*, rev |
| AS144 | CTAGATGGCGCGTTACCTGGTAGCGCGCCATTTTGTTTT | T*crp*, fwd |
| AS145 | CTAGAAAACAAAATGGCGCGCTACCAGGTAACGCGCCAT | T*crp*, rev |
| AS147 | GACAGCCT*CAC*TGGCA**CCTCTAGAAAACAAAATGGC** | Fragment 2F-T*crp*, down**^b^** |
| AS153 | GACAGCCT*CAC*TGGCA**CCTCTAGACAAAAGCCTCCG** | Fragment 2F-T*tonB*, down**^b^** |
| AS159 | **TCCTCCTTAGTTCCTATTCC** | Integration of P_tetO1_-*cI* into *folD*, *cam* up |
| AS160 | ACTTCGGAATAGGAACTAAGGAGGA**AAATAGGCGTATCACGAGGC** | Integration of P_tetO1_-*cI* into *folD*, *cI* up |
| AS161 | CATCAATAATAAGGCTTTATGCTAGATGCATTCCGCTTTGCGACTCAACC**ACTAGCAACACCAGAACAGC** | Integration of networks into att_HK022_, up |
| AS167 | CATCCAGAGTCTTCGGGTCAGGGTTAAATTCACGGTCGGTGCACTTTAG**GGCTTACCCGTCTTACTGTCC** | Integration of networks into att_HK022_, down |
| AS168 | CACCGTCGCTGAGACTGAAAGCTTCATTTTTCGTCCATGATGGCGTTGTA**GAAAAGTGCCACCTGCATCG** | Integration of P_LtetO1_-*cI* into *folD*, *cam* down |
| AS170 | TTCTTAAATTATCTTAATCCTTAGACAAGGAAATAAATCAGTTCCAGATT**TAGATCAAGCTGCTAAAGCG** | Integration of P_LtetO1_-*cI* into *folD*, *cI* down |
| AS171 | **CATCCCTATCAGTGATAGAG**C**TAC**G**GAGCACATCAGCAGGACGC** | A-11C and T-7G substitution, fwd |
| AS172 | **GCGTCCTGCTGATGTGCTC**C**GTA**G**CTCTATCACTGATAGGGATG** | A-11C and T-7G substitution, rev |
| AS195 | GACAGCCT*AGG*TGGC**ATTAAAGAGGAGAAAGGTACC** | Promoterless *yfp*, fwd |
| AS198 | ATTAgcatgc**TAATAGGTATCCTATGATTA** | wt P*_lac_*, fwd |
| AS199 | TAATgaattc**TCCTTCTCGATCCGAGACGA** | wt P*_lac_*, rev |
| AS206 | CATTAATGCAGCTGGCACGACAGGTTTCCCGACTGGAAAGCGGGCAGTGA**CATATGAATATCCTCCTTAG** | Integration of Cam^R^ into P*_lac_*, fwd |
| AS207 | AAGCCTGGGGTGCCTAATGAGTGAGCTAACTCACATTAATTGCGTTGCGC**ACAGCTGCAGGCATGCAAGC** | Integration of Cam^R^ into P*_lac_*, rev |
| AS221 | TAAAACTATCAGCCAGGTCATTATCGCCTGGCTGATTTTTAGCTTACTGT**CACCATCGAATGGCGCAAAA** | Integration of *lacI* into *yeaH* locus, fwd |
| AS222 | TCATATTTAAAGCGATTGTAAGCTAATGTATGTAATAAATGAGATAATTT**ACAGCTGCAGGCATGCAAGC** | Integration of *lacI* into *yeaH* locus, rev |
| AS225 | CATCTTAAGCGCCCTCGACCTTTATGGTTGAGGGCGTTTTGCTATGAACG**CACCATCGAATGGCGCAAAA** | Integration of *lacI* into *asnT* locus, fwd |
| AS226 | GAGACTACTGAATAACTCAAGTTTTATAATCGAGGGGAAAATGGTGATGG**ACAGCTGCAGGCATGCAAGC** | Integration of *lacI* into *asnT* locus, rev |
| AS229 | GCAACATTCCAGCAGCGGTAACGACGTACCGCTGCTTTTTTTTGCCCCAA**CACCATCGAATGGCGCAAAA** | Integration of *lacI* into *flhC* locus, fwd |
| AS230 | TCCACTGTTGACCATGACAGGATGTTCAGTCGTCAGGCGTTAACGCGCGA**ACAGCTGCAGGCATGCAAGC** | Integration of *lacI* into *flhC* locus, rev |
| AS267 | TTGTCGGCGGTGGTGATGTC | qPCR *cysG*, up |
| AS268 | ATGCGGTGAACTGTGGAATAAACG | qPCR *cysG*, down |
| AS271 | CGTGCACATCAGACATTGTG | *yeaH-lacI* junction, fwd |
| AS272 | CGTTTTCGCAGAAACGTGGC | *lacI* junctions, rev |
| AS277 | GCCTGCAGCTTATGTCAACC | *flhC-lacI* junction, fwd |
| AS280 | GTCACTGACCTTAGTTGAAC | *asnT-lacI* junction, fwd |
| AS282 | GACAGCCT*CAC*TGGCT**CTAGGGaagagtttgt** | Fragment 2F-T1T2, down**^b^** |
| AS283 | TCGACTCTAGATGGCGCGTTACCTGGTAGCGCGCCATTTTGTTTTCTAGAGGTGCCAGTGAGGCACCTTTCGTCTTCACCTCGAGG | T*crp*, fwd |
| AS284 | TCGACCTCGAGGTGAAGACGAAAGGTGCCTCACTGGCACCTCTAGAAAACAAAATGGCGCGCTACCAGGTAACGCGCCATCTAGAG | T*crp*, rev |
| AS285 | TCGACTCTAGAAAAAGCCTCCGACCGGAGGCTTTTGTCTAGAGGTGCCAGTGAGGCACCTTTCGTCTTCACCTCGAGG | T*tonB*, fwd |
| AS286 | TCGACCTCGAGGTGAAGACGAAAGGTGCCTCACTGGCACCTCTAGACAAAAGCCTCCGGTCGGAGGCTTTTTCTAGAG | T*tonB*, rev |
| KTp38 | **TCAGTGATAGAGATTGACATCCCT** | RNA purity, *cI* up |
| KTp39 | **CCCCACAACGGAACAACTCT** | RNA purity, *cI* down |
| KTp45 | GACAGCCT*CAC*TGGCTC**TTAAGCTGCTAAAGCGTAG** | Fragment 2 without T1, down**^b^** |
| KTp46 | ATTCGCCA*TTC*AGGCTC**TTAAGCTGCTAAAGCGTAG** | Fragment 2R without T1, up**^b^** |
| KTp65 | **GCTGTTGAGCCAGGTGATTT** | qPCR *cI*, up |
| KTp66 | **GGGATCATTGGGTACTGTGG** | qPCR *cI*, down |
| KTp67 | **AATACGCAAACCGCCTCTC** | qPCR *lacI*, up |
| KTp68 | **CAGTCGGGAAACCTGTCGT** | qPCR *lacI*, down |
| KTp71 | **GTTGTCACTGAAGCGGGAAG** | qPCR *kanR*, up |
| KTp72 | **GCAAGGTGAGATGACAGGAGA** | qPCR *kanR*, down |
| KTp73 | **ACTCATCACCCCCAAGTCTG** | Northern blot, *cI* probe 1 |
| KTp74 | **GGATCATTGGGTACTGTGGG** | Northern blot, *cI* probe 2 |
| KTp75 | **CCTGACTGCCCCATCCCC** | Northern blot, *cI* probe 3 |
| KTp76 | **CTCGTCCTGCAGTTCATTCA** | Northern blot, *kanR* probe 1 |
| KTp77 | **GCCAACGCTATGTCCTG** | Northern blot, *kanR* probe 2 |
| KTp90 | ATGGATCCT**ATTAAGCTGCTAAAGC** | P_LlacO1_-*tetR/lacI*, down |
| KTp91 | ATGCATGC**TCGAGAATTGTGAGC** | P_LlacO1_-*tetR/lacI*, up |
| KTp93 | **CGGTTTGCGTATTGGGCG** | Northern blot, *lacI* probe 1 |
| KTp94 | **AGAAGATTGTGCACCGCC** | Northern blot, *lacI* probe 2 |
| 5’_recA | TGACTATCCGGTATTACCCGGCATGACAGGAGTAAAAATG**GGGGATCCGTCGACCTGCAGTT** | *recA* deletion, up |
| 3’_recA | AAGGGCCGCAGATGCGACCCTTGTGTATCAAACAAGACGA**TGTAGGCTGGAGCTGCTTC** | *recA* deletion, down |

^a^ Restriction sites are underlined; sequences of overhangs produced after restriction with BglI are italicized, annealing sequences are shown in bold.

^b^ Fragment number refers to gene position in the three gene array; F stands for “forward” orientation (opposite to the orientation of the kanamycin cassette), R for “reverse” orientation.
