# Supplementary material for: Local genetic context shapes the function of a gene regulatory network
Source: eLife. 2021 Mar 8;10:e65993. doi: 10.7554/eLife.65993 (PMC7968929; doi:10.7554/eLife.65993)
Supplement: Supplementary file 3. [file elife-65993-supp3.docx]

**Supplementary File 3. Plasmids used in this study.**

| **Name** | **Description^a^** | **Primers and enzymes used for cloning** | **Source** |
| --- | --- | --- | --- |
| pAS014 | *pSC101* ori*, Amp^R^, P_LlacO1_-*tetR* (T) | AS104/AS105; XhoI+XbaI | This study |
| pAS015 | *pSC101* ori*, Amp^R^, P_LlacO1_-*lacI* (L) | AS104/AS105; XhoI+XbaI | This study |
| pAS016 | *pSC101* ori*, Amp^R^, P_LtetO1_-*cI* (C) | AS106/AS107; XhoI+XbaI | This study |
| pAS017 | *R6K* ori, Kan^R^, *attP_λ_*, P*_lac_*-*mCherry* | AS096/AS097; PstI+SmaI | This study |
| pAS019 | *pSC101* ori*, Kan^R^, *mCherry* | AS135/AS136; ScaI+EcoRI | This study |
| pAS020 | *pSC101* ori*, Amp^R^, P_LlacO1_-*lacI*-T*crp*-T1 | AS142/AS143; XbaI | This study |
| pAS021 | *pSC101* ori*, Amp^R^, P_LlacO1_-*lacI*-T*tonB*-T1 | AS144/AS145; XbaI | This study |
| pAS022 | *R6K* ori, Cam^R^-*frt*, *attP_λ_*, P_LlacO1_-*tetR* (from pKT11) | BamHI+SphI | This study |
| pAS023 | *pSC101* ori*, Amp^R^, P_LtetO1(A-11C, T-7G)_-*cI* | AS171/AS172 | This study |
| pAS024 | *pSC101* ori*, Amp^R^, P_LtetO1(A-11C, T-7G)_-*yfp* | AS171/AS172 | This study |
| pAS026 | *pSC101* ori*, Kan^R^, P_LlacO1_-*tetR*-P_LlacO1_-*lacI-*P_LtetO1(A-11C, T-7G)_-*cI* | AS098/AS099, AS100/AS101, AS102/AS103; BglI | This study |
| pAS035 | *pSC101* ori*, Kan^R^, *yfp*_rev_ | AS129/AS195; BglI | This study |
| pAS036 | *pSC101* ori*, Kan^R^, P_LtetO1_-*yfp* | AS098/AS103; BglI | This study |
| pAS037 | *pSC101* ori*, Kan^R^, P_LtetO1_-*yfp*_rev_ | AS129/AS134; BglI | This study |
| pAS038 | *pSC101* ori*, Amp^R^, P_LlacO1_-*lacI*-T1T2 | XbaI | This study |
| pAS039 | *pSC101* ori*, Kan^R^, P_LlacO1_-*tetR*-P_LlacO1_-*lacI-T1T2-*P_LtetO1_-*cI* | AS098/AS099, AS100/AS282, AS102/AS103; BglI | This study |
| pAS040 | *pSC101* ori*, Kan^R^, P_LlacO1_-*lacI-*T*crp-*T1-P_LlacO1_-*lacI-*P_LtetO1_-*cI* | AS098/AS099, AS100/AS101, AS102/AS103; BglI | This study |
| pAS041 | *pSC101* ori*, Kan^R^, T1-*yfp* | SalI | This study |
| pAS042 | *pSC101* ori*, Kan^R^, T*crp*-*yfp* | AS283/AS284;SalI | This study |
| pAS043 | *pSC101* ori*, Kan^R^, T*tonB*-*yfp* | AS285/AS286;SalI | This study |
| pAS045 | *pSC101* ori*, Kan^R^, P_LlacO1_-*lacI*-P_LtetO1(A-11C,T-7G)_-*cI*-P_LlacO1_-*tetR* | AS098/AS099, AS100/AS101, AS102/AS103; BglI | This study |
| pAS046 | *pSC101* ori*, Kan^R^, P_LlacO1_-*tetR_r_*-P_LtetO1(A-11C,T-7G)_-*cI_r_-*P_LlacO1_-*lacI_r_* | AS129/AS130, AS131/AS132, AS133/AS134; BglI | This study |
| pAS047 | *pSC101* ori*, Kan^R^, P_LlacO1_-*tetR*-P_LtetO1(A-11C,T-7G)_-*cI-*P_LlacO1_-*lacI* | AS098/AS099, AS100/AS101, AS102/AS103; BglI | This study |
| pAS050 | *pSC101* ori*, Kan^R^, P_LtetO1(A-11C,T-7G)_-*cI_r_-*P_LlacO1_-*lacI_r_-*PLlacO1-*tetR_r_* | AS129/AS130, AS131/AS132, AS133/AS134; BglI | This study |
| pAS051 | *pSC101* ori*, Kan^R^, P_LlacO1_-*lacI_r_-*P_LtetO1(A-11C,T-7G)_-*cI_r_-* PLlacO1-*tetR_r_* | AS129/AS130, AS131/AS132, AS133/AS134; BglI | This study |
| pAS053 | *pSC101* ori*, Kan^R^, P_LlacO1_-*tetR*-P_LlacO1_-*lacI-*T*crp-*P_LtetO1_-*cI* | AS098/AS099, AS100/AS147, AS102/AS103; BglI | This study |
| pAS055 | *pSC101* ori*, Kan^R^, P_LlacO1_-*tetR*-P_LlacO1_-*lacI-*T*tonB-*P_LtetO1_-*cI* | AS098/AS099, AS100/AS153, AS102/AS103; BglI | This study |
| pCC01 | *R6K* ori, Cam^R^-*frt*, *attP*_λ_, P*_lac_*-*yfp* | AS198/AS199; EcoRI+SphI | This study |
| pKT10 | *pSC101* ori*, Kan^R^ | XhoI+SalI | This study |
| pKT11 | *R6K* ori, Cam^R^-*frt*, *attP_P21_*, P_LlacO1_-*tetR* | KTp90/KTp91; BamHI+SphI | This study |
| pKT12 | *R6K* ori, Cam^R^-*frt*, *attP_HK022_*, P_LlacO1_-*lacI* | KTp90/KTp91; BamHI+SphI | This study |
| D052 | *pSC101* ori*, Amp^R^, P_LlacO1_-*lacI_f_*-P_LtetO1_-*cI-*P_LlacO1_-*lacI-*P_R_-*gfp* |  | (Guet et al., 2002) |
| pN1 | *pSC101* ori*, Kan^R^, P_LtetO1_-*cI*-P_LlacO1_-*lacI-*P_LlacO1_-*tetR* (CLT) | AS098/AS099, AS100/AS101, AS102/AS103; BglI | This study |
| pN2 | *pSC101* ori*, Kan^R^, P_LtetO1_-*cI*-P_LlacO1_-*tetR-*P_LlacO1_-*lacI* (CTL) |  | This study |
| pN3 | *pSC101* ori*, Kan^R^, P_LlacO1_-*lacI*-P_LtetO1_-*cI-*P_LlacO1_-*tetR* (LCT) |  | This study |
| pN4 | *pSC101* ori*, Kan^R^, P_LlacO1_-*lacI*-P_LlacO1_-*tetR-*P_LtetO1_-*cI* (LTC) |  | This study |
| pN5 | *pSC101* ori*, Kan^R^, P_LlacO1_-*tetR-*P_LtetO1_-*cI-*P_LlacO1_-*lacI* (TCL) |  | This study |
| pN6 | *pSC101* ori*, Kan^R^, P_LlacO1_-*tetR*-P_LlacO1_-*lacI-*P_LtetO1_-*cI* (TLC) |  | This study |
| pN9 | *pSC101* ori*, Kan^R^, P_LlacO1_-*lacI*-P_LtetO1_-*cI_r_-*P_LlacO1_-*tetR* (LC_r_T) | AS098/AS099, KTp46/AS132, AS102/AS103; BglI | This study |
| pN10 | *pSC101* ori*, Kan^R^, P_LlacO1_-*lacI*-P_LlacO1_-*tetR_r_-*P_LtetO1_-*cI* (LT_r_C) |  | This study |
| pN11 | *pSC101* ori*, Kan^R^, P_LlacO1_-*tetR*-P_LtetO1_-*cI_r_-*P_LlacO1_-*lacI* (TC_r_L) |  | This study |
| pN12 | *pSC101* ori*, Kan^R^, P_LlacO1_-*tetR*-P_LlacO1_-*lacI_r_-*P_LtetO1_-*cI* (TL_r_C) |  | This study |
| pN13 | *pSC101* ori*, Kan^R^, P_LtetO1_-*cI*-P_LlacO1_-*lacI-*P_LlacO1_-*tetR_r_* (CLT_r_) | AS098/AS099, AS100/KTp45, AS133/AS134; BglI | This study |
| pN14 | *pSC101* ori*, Kan^R^, P_LtetO1_-*cI*-P_LlacO1_-*tetR-*P_LlacO1_-*lacI_r_* (CTL_r_) |  | This study |
| pN16 | *pSC101* ori*, Kan^R^, P_LlacO1_-*lacI*-P_LlacO1_-*tetR-*P_LtetO1_-*cI_r_* (LTC_r_) |  | This study |
| pN18 | *pSC101* ori*, Kan^R^, P_LlacO1_-*tetR*-P_LlacO1_-*lacI-*P_LtetO1_-*cI_r_* (TLC_r_) |  | This study |
| pN19 | *pSC101* ori*, Kan^R^, P_LtetO1_-*cI*-P_LlacO1_-*lacI_r_-*P_LlacO1_-*tetR_r_* (CL_r_T_r_) | AS098/AS099, KTp46/AS132, AS133/AS134; BglI | This study |
| pN20 | *pSC101* ori*, Kan^R^, P_LtetO1_-*cI*-P_LlacO1_-*tetR_r_-*P_LlacO1_-*lacI_r_* (CT_r_L_r_) |  | This study |
| pN21 | *pSC101* ori*, Kan^R^, P_LlacO1_-*lacI*-P_LtetO1_-*cI_r_-*P_LlacO1_-*tetR_r_* (LC_r_T_r_) |  | This study |
| pN22 | *pSC101* ori*, Kan^R^, P_LlacO1_-*lacI*-P_LlacO1_-*tetR_r_-*P_LtetO1_-*cI_r_* (LT_r_C_r_) |  | This study |
| pN23 | *pSC101* ori*, Kan^R^, P_LlacO1_-*tetR*-P_LtetO1_-*cI_r_-*P_LlacO1_-*lacI_r_* (TC_r_L_r_) |  | This study |
| pN24 | *pSC101* ori*, Kan^R^, P_LlacO1_-*tetR*-P_LlacO1_-*lacI_r_-*P_LtetO1_-*cI_r_* (TL_r_C_r_) |  | This study |
| pN25 | *pSC101* ori*, Kan^R^, P_LtetO1_-*cI_r_*-P_LlacO1_-*lacI_r_-*P_LlacO1_-*tetR_r_* (C_r_L_r_T_r_) | AS129/AS130, AS131/AS132, AS133/AS134; BglI | This study |
| pN26 | *pSC101* ori*, Kan^R^, P_LtetO1_-*cI_r_*-P_LlacO1_-*tetR_r_-*P_LlacO1_-*lacI_r_* (C_r_T_r_L_r_) |  | This study |
| pN27 | *pSC101* ori*, Kan^R^, P_LlacO1_-*lacI_r_*-P_LtetO1_-*cI_r_-*P_LlacO1_-*tetR_r_* (L_r_C_r_T_r_) |  | This study |
| pN28 | *pSC101* ori*, Kan^R^, P_LlacO1_-*lacI_r_*-P_LlacO1_-*tetR_r_-*P_LtetO1_-*cI_r_* (L_r_T_r_C_r_) |  | This study |
| pN29 | *pSC101* ori*, Kan^R^, P_LlacO1_-*tetR_r_*-P_LtetO1_-*cI_r_-*P_LlacO1_-*lacI_r_* (T_r_C_r_L_r_) |  | This study |
| pN30 | *pSC101* ori*, Kan^R^, P_LlacO1_-*tetR_r_*-P_LlacO1_-*lacI_r_-*P_LtetO1_-*cI_r_* (T_r_L_r_C_r_) |  | This study |
| pN33 | *pSC101* ori*, Kan^R^, P_LlacO1_-*lacI_r_*-P_LtetO1_-*cI_r_-*P_LlacO1_-*tetR* (L_r_C_r_T) | AS129/AS130, AS131/AS132,  AS102/AS103; BglI | This study |
| pN34 | *pSC101* ori*, Kan^R^, P_LlacO1_-*lacI_r_*-P_LlacO1_-*tetR_r_-*P_LtetO1_-*cI* (L_r_T_r_C) |  | This study |
| pN35 | *pSC101* ori*, Kan^R^, P_LlacO1_-*tetR_r_*-P_LtetO1_-*cI_r_-*P_LlacO1_-*lacI* (T_r_C_r_L) |  | This study |
| pN36 | *pSC101* ori*, Kan^R^, P_LlacO1_-*tetR_r_*-P_LlacO1_-*lacI_r_-*P_LtetO1_-*cI* (T_r_L_r_C) |  | This study |
| pN37 | *pSC101* ori*, Kan^R^, P_LtetO1_-*cI_r_*-P_LlacO1_-*lacI-*P_LlacO1_-*tetR* (C_r_LT) | AS129/AS130, AS100/AS101, AS102/AS103; BglI | This study |
| pN39 | *pSC101* ori*, Kan^R^, P_LlacO1_-*lacI_r_*-P_LtetO1_-*cI-*P_LlacO1_-*tetR* (L_r_CT) |  | This study |
| pN41 | *pSC101* ori*, Kan^R^, P_LlacO1_-*tetR_r_*-P_LtetO1_-*cI-*P_LlacO1_-*lacI* (T_r_CL) |  | This study |
| pN43 | *pSC101* ori*, Kan^R^, P_LtetO1_-*cI_r_*-P_LlacO1_-*lacI-*P_LlacO1_-*tetR_r_* (C_r_LT_r_) | AS129/AS130, AS100/KTp45, AS133/AS134; BglI | This study |
| pN44 | *pSC101* ori*, Kan^R^, P_LtetO1_-*cI_r_*-P_LlacO1_-*tetR-*P_LlacO1_-*lacI_r_* (C_r_TL_r_) |  | This study |
| pN45 | *pSC101* ori*, Kan^R^, P_LlacO1_-*lacI_r_*-P_LtetO1_-*cI-*P_LlacO1_-*tetR_r_* (L_r_CT_r_) |  | This study |
| pN47 | *pSC101* ori*, Kan^R^, P_LlacO1_-*tetR_r_*-P_LtetO1_-*cI-*P_LlacO1_-*lacI_r_* (T_r_CL_r_) |  | This study |
| pN49 | *pSC101* ori*, Kan^R^, P_LtetO1_-*yfp*-P_LlacO1_-*lacI-*P_LlacO1_-*tetR* (VLT) | AS098/AS099, AS100/AS101, AS102/AS103; BglI | This study |
| pN54 | *pSC101* ori*, Kan^R^, P_LlacO1_-*tetR*-P_LlacO1_-*lacI-*P_LtetO1_-*yfp* (TLV) |  | This study |
| pZS*12-*gfp* | *pSC101* ori*, Amp^R^, P_LlacO1_-*gfp* |  | Laboratory stock |
| pZS*2R-*gfp* | *pSC101* ori*, Kan^R^, P_R_-*gfp* |  | Laboratory stock |
| pZS*11-*yfp* | *pSC101* ori*, Amp^R^, P_LtetO1_-*yfp* |  | Laboratory stock |
| pZA31-CFP | *P15A ori*, Cam^R^, P_LtetO1_-*cfp* |  | Laboratory stock |
| pLA2 | *R6K ori*, Kan^R^, *attP_λ_* |  | (Haldimann and Wanner, 2001) |
| pBS3Clux | *pMB1 ori*, Amp^R^, P*_lac_*-*mCherry*, *luxABCDE* |  | (Radeck et al., 2013) |
| pKD3 | *R6K ori*, Cam^R^-*frt*, Amp^R^ |  | (Datsenko and Wanner, 2000) |
| pKD13 | *R6K ori*, Kan^R^-*frt*, Amp^R^ |  | (Datsenko and Wanner, 2000) |
| pAH68-frt-cat | *R6K* ori, Cam^R^-*frt*, *attP_HK022_* |  | (Pleška et al., 2016) |
| pAH120-frt-cat | *R6K* ori, Cam^R^-*frt*, *attP_λ_* |  | (Pleška et al., 2016) |

^a^ Amp – ampicillin resistance, Kan – kanamycin resistance, Cam – chloramphenicol resistance
