# Supplementary material for: Local genetic context shapes the function of a gene regulatory network
Source: eLife. 2021 Mar 8;10:e65993. doi: 10.7554/eLife.65993 (PMC7968929; doi:10.7554/eLife.65993)
Supplement: Supplementary file 4. [file elife-65993-supp4.docx]

**Supplementary File 4. ANOVA test statistics.**

| **IPTG [mM]** | **F(DFn, DFd)** | **P value** |
| --- | --- | --- |
| 0 | F_2,24_=1.957 | P=0.1632 |
| 0.1 | F_2,24_=14.87 | P<0.0001 |
| 0.2 | F_2,24_=13.08 | P=0.0001 |
| 0.3 | F_2,18_=22.53 | P<0.0001 |
| 0.4 | F_2,21_=7.345 | P=0.0038 |
| 1 | F_2,24_=4.500 | P=0.0219 |
